# Hepatic loss of CerS2 induces cell division defects via a mad2‐mediated pathway

**DOI:** 10.1002/ctm2.712

**Published:** 2022-01-28

**Authors:** Mingjun Cao, Shaohua Zhang, Sin Man Lam, Guanghou Shui

**Affiliations:** ^1^ State Key Laboratory of Molecular Developmental Biology, Institute of Genetics and Developmental Biology Chinese Academy of Sciences Beijing China; ^2^ University of Chinese Academy of Sciences Beijing China; ^3^ Lipidall Technologies Company Limited Changzhou Jiangsu Province China

Cytokinesis failure is the primary cause of hepatocyte polyploidization.^1^ However, there are few studies on ceramide synthase and polyploidy. Ceramide synthases (CerS) have six isoforms (CerS1‐CerS6), each of which can synthesize ceramides with different acyl chain lengths (C14:0‐C30:0) and possess tissue‐specific distribution.^2^, ^3^, ^4^ Ceramide synthase 2 (CerS2) preferentially synthesizes ceramides with longer acyl chains of C22 and C24.^5^, ^6^ In human hepatocellular carcinoma (HCC), CerS2 gene expression was lower compared with the normal liver.^7^ We found that the deletion of CerS2 led to abnormal hepatic chromosome polyploidy and substantial steatosis and hepatic carcinoma in 15‐month‐old mice. Further studies demonstrated that CerS2 plays a critical role in maintaining hepatic chromosome polyploidization via Mad2 expression during cell division.

First, using the Cre‐loxp system, we generated a liver‐specific CerS2 gene knockout mouse (cKO mice) (Figure 1A–G). To determine whether CerS2 deficiency affects liver physiology, haematoxylin and eosin (H&E) staining was performed. The hepatocytes from cKO mice at 3 weeks (before weaning), 1 month, 3 months (after weaning) and 6 months exhibited abnormally enlarged nuclei (Figure 2A,B). Next, flow‐cytometric analyses on cell cycle profiles demonstrated that the CerS2‐depleted hepatocytes displayed a significantly higher proportion of octoploid or hexadecploid hepatocytes and fewer diploid hepatocytes than the WT controls (Figure 2C). Further analysis showed that the proportion of octoploid hepatocytes in cKO mice gradually increased with age (Figure 2D). To determine the number of nuclei in each hepatocyte, the nuclear and cell membrane (β‐catenin) of the liver sections were stained (Figure 2E,F). The results demonstrated that the depletion of CerS2 in the livers of same‐aged mice led to strong induction of mononucleated polyploid hepatocytes with enlarged nuclei compared to wild‐type mice.

**FIGURE 1:**
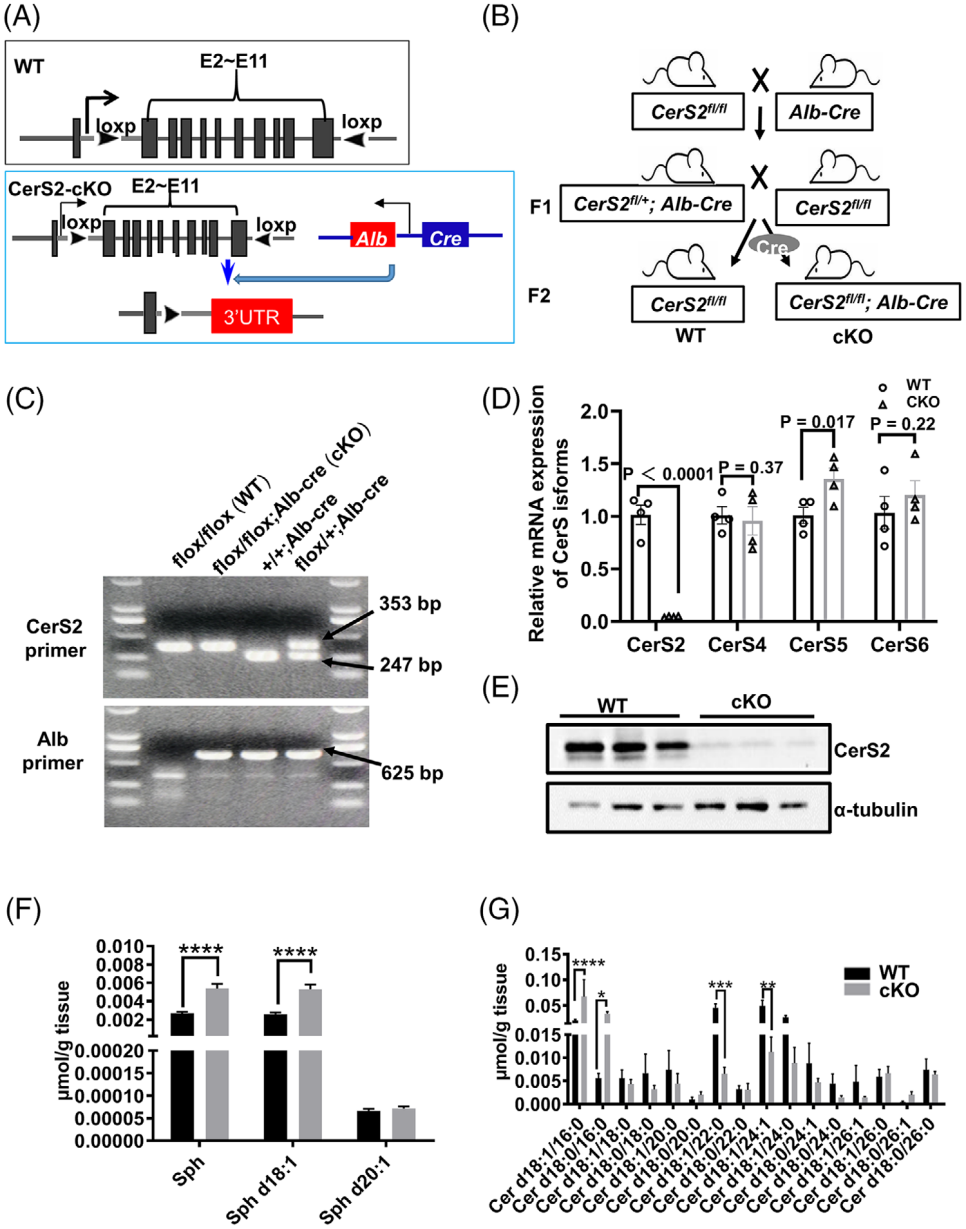
Generation of CerS2 conditional mice. (A) WT represents the addition of loxP sites in exons 2 to 11, and CerS2‐cKO shows the mutation sequence of the CerS2 gene after crossing WT mice with Alb‐Cre mice. (B) The breeding program of CerS2 liver‐specific knockout mice. (C) Genotyping PCR analyses performed with tail tip genomic DNA (gDNA) from WT, heterozygous and homozygous mice. PCR was performed to validate the presence of the 5′loxP site and the Alb fragment. Expected DNA fragments: WT has bands at 247 bp, and cKO has bands at 353 and 625 bp. (D) Relative mRNA expression of CerS isoforms in WT and CerS2‐KO mice liver issues. CerS isoforms (CerS2, CerS4, CerS5, CerS6) (E) Western blot analyses of CerS2 expression in the liver of CerS2‐WT, and ‐cKO mice normalized to α‐tubulin. *n* = 3 per group. (F, G) The levels of sphinganine (Sph) and ceramide (Cer) in cKO mice measured by LC‐MS/MS. *n* = 4 per group. *p < .05; **p < .01; ***p < .001 and ****p < .0001

**FIGURE 2:**
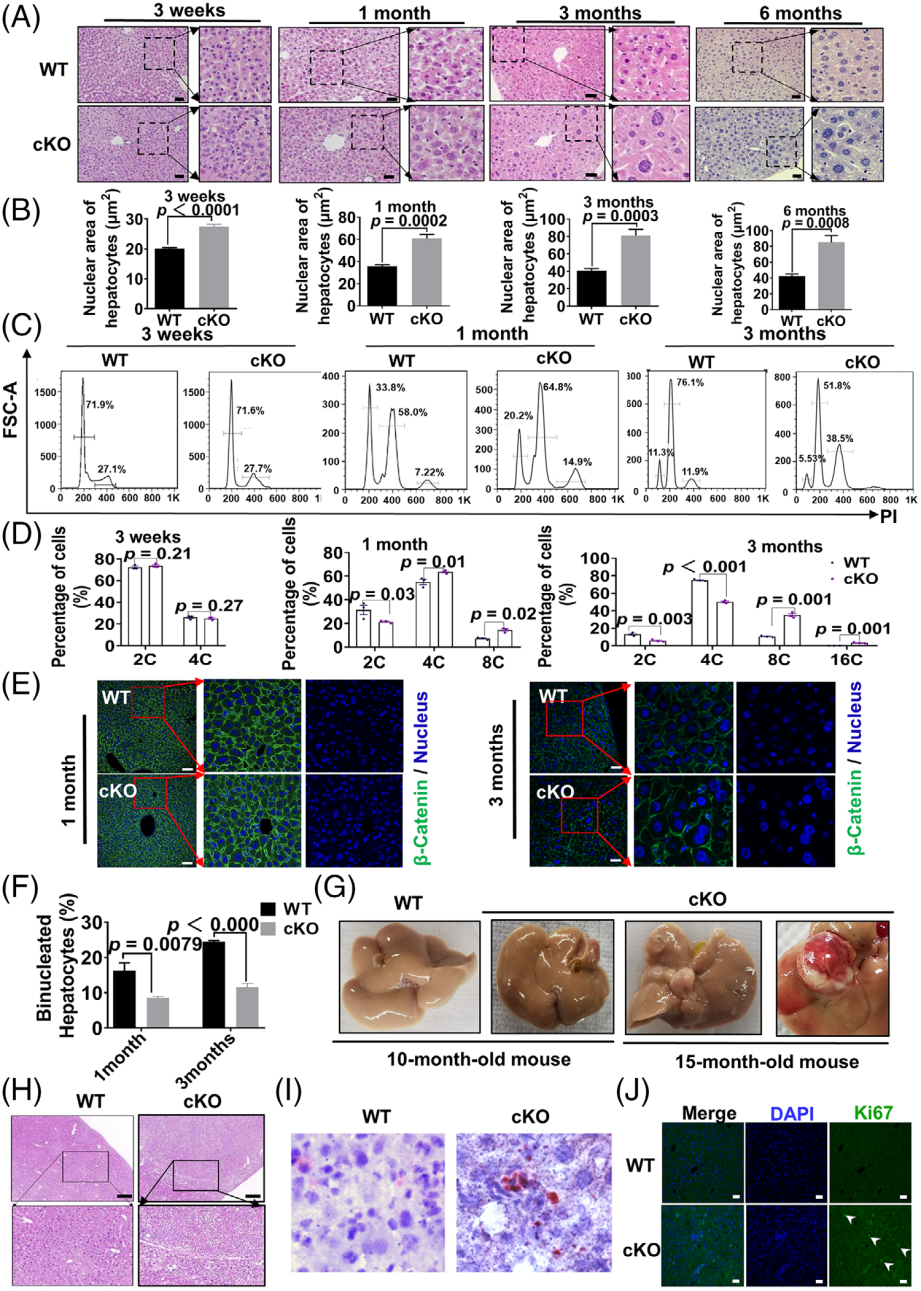
CerS2 is involved in the regulation of hepatic chromosome polyploidization and hepatocellular carcinoma. (A) Representative images of haematoxylin and eosin (H&E)‐stained liver sections of WT and cKO mice at 3 weeks, 1 month, 3 months and 6 months. The black dotted area on the left indicates magnification. Scale bars: 50 μm. (B) Statistics of the hepatocyte nucleus area of liver tissue from 3 weeks, 1 month, 3 months and 6‐months. (C) The genome ploidies of hepatocytes were characterized by propidium iodide staining, followed by fluorescence‐activated cell sorting. Hepatocytes were isolated from WT and cKO mice at 1 month and 3 months of age. (D) Quantification of DNA contents isolated from hepatocytes of 3 weeks, 1 month and 3 months WT and cKO mice. (E) Representative images of immunofluorescence staining for β‐catenin (plasma membrane, green) and nuclei (blue) in liver sections of 1‐ and 3‐month‐old WT and cKO mice; scale bars: 50 μm. (F) Quantification of binucleated hepatocytes in liver sections of 1‐ and 3‐month‐old WT and cKO mice. (G) The liver on the left is from a 10‐month WT and cKO mouse. The liver on the right is from a 15‐month WT and cKO mouse. At 10 months of age, the liver capsular surface began to appear abnormal, while around 15 months different sizes of coloured nodules appeared. (H) The images of H&E‐stained liver sections of WT and cKO mice at 15 months. The black dotted area on the left indicates magnification. Scale bars: 100 μm. (I) Representative images of hepatic ORO staining of mice at 15 months. (J) Representative images of immunofluorescence staining for Ki‐67 in liver sections from 15‐month‐old WT and cKO mice; scale bars: 50 μm. *n* = 4 or 6 per group. Data are presented as the means ± SD. FSC, forward scatter

As mice grew, we observed that the surface of the liver capsular showed randomly scattered nodules of variable sizes and colours, indicating focal fat accumulation (Figure 2G). Results of H&E staining and oil red staining showed CerS2 deficiency could potentially induce hepatic steatosis‐like pathology (Figure 2H,I). Meanwhile, a significant expression of Ki‐67 in cKO mice indicated that CerS2 deficiency‐induced abnormal chromosome polyploidy may progress to hepatic carcinoma with the age of mice (Figure 2J).

The cell nucleus is an organelle highly capable of lipid metabolism^8^ and is involved in many cellular processes. We investigated whether CerS2 deficiency affects sphingolipid metabolism in isolated hepatocyte nuclei (Figure S1A). LC‐MS/MS analysis^9^ showed that there was no difference in the contents of individual classes in cKO mice, while sphingolipids with specific acyl chain lengths showed varying degrees of disorder (Figure S1B–D).

To explore the mechanism of abnormal chromosome polyploidy in CerS2‐cKO mice, immunostaining on S‐phase marker PCNA and the phosphorylation of histone H3^S10^ was performed to determine the cell cycle expression pattern of the hepatocytes (Figure 3A,B). We observed that PCNA‐positive proportions were slightly higher in cKO livers at 3 weeks, while there was no significant difference in the proportions of p‐HH3^S10^‐positive nuclei (Figure 3A,B). For 1‐month‐old mice, the number of PCNA‐positive hepatocytes in cKO mice was approximately four times higher than that of WT mice, while the number of p‐HH3^S10^‐positive hepatocytes was only two times higher than that of WT mice (Figure 3A,B). These results indicate that a high population of polyploid cells in cKO livers was arrested in mitosis rather than the S phase.

**FIGURE 3:**
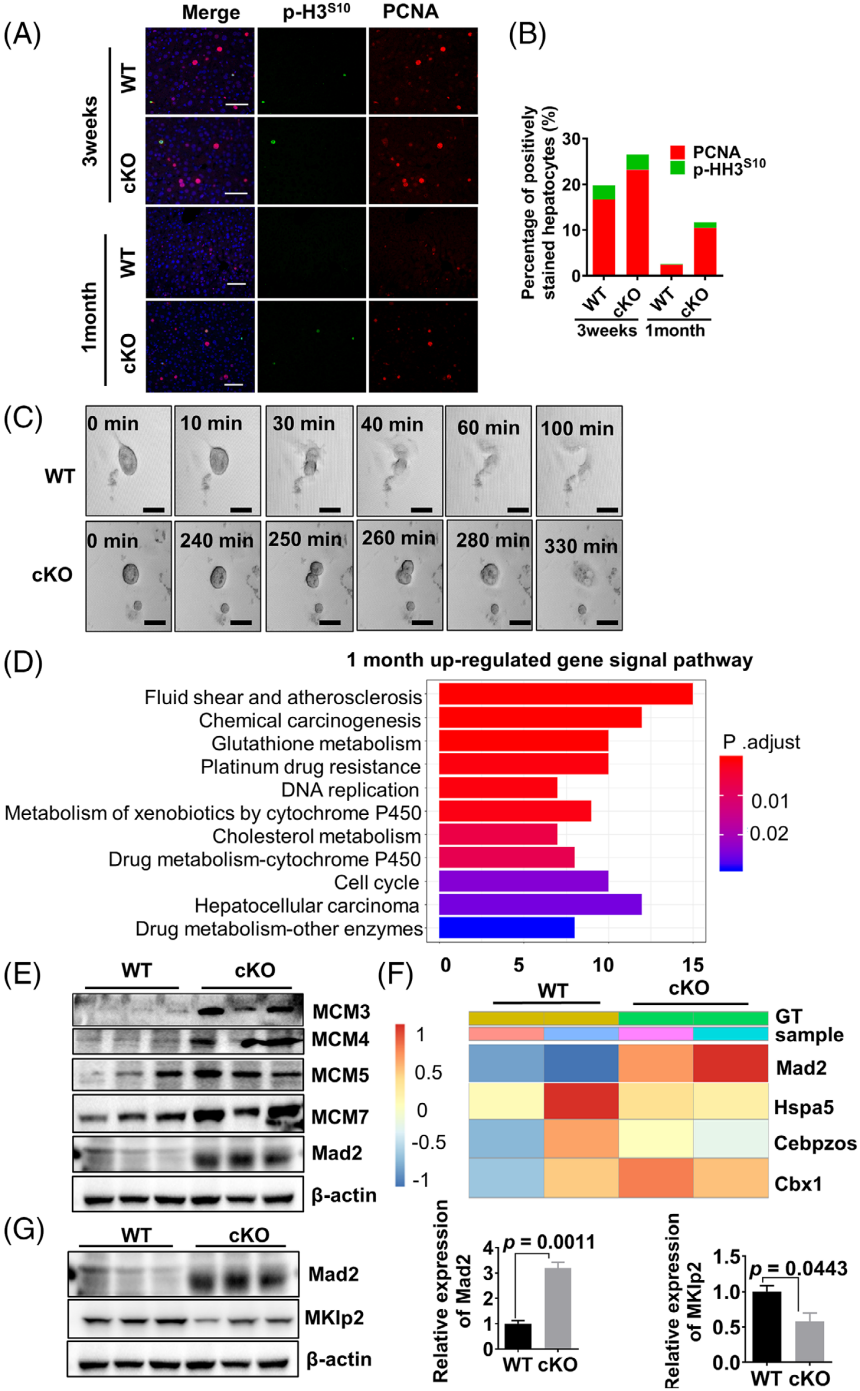
Depletion of CerS2 causes hepatocellular division defects. (A) Double immunofluorescence staining for PCNA (red) and phospho‐H3^S10^ (green) expression and nuclei (blue) in the liver sections of WT and cKO mice at 3 weeks and 1 month of age. Scale bars: 50 μm. (B) Quantification of positively stained hepatocytes in liver sections. PCNA (red), a marker of the S phase; phospho‐H3^S10^ (green), a marker of mitosis. *n* = 4 mice per group. (C) Time‐lapse imaging to analyse the cell cycle of primary hepatocytes in WT and cKO mice at 1 month of age. Results are representative of three independent experiments. Scale bars: 25 μm. (D) Gene expression signal pathway representing fold‐change ratios of hepatocytes from cKO mice versus WT mice at 1 month of age. *n* = 2 mice per group. (E) Expression of the cell cycle‐related gene analysis by immunoblotting of hepatocytes from WT and cKO mice. *n* = 3 mice per group. (F) Partial gene expression heat map representing fold‐change ratios of hepatocytes from cKO mice versus WT mice at 1 month of age. *n* = 2 mice per group. (G) Statistical analysis of Mad2 and Mklp2 expression proteins. *n* = 3 mice per group. Results are presented as means ± standard error of the means

To further determine which stage of mitosis of cKO hepatocytes was arrested, time‐lapse imaging was used to visualize the cell cycle. As displayed in Figure 3C, the mitotic metaphase of CerS2‐deficient hepatocytes was significantly delayed, indicating that depletion of CerS2 caused hepatocellular division defects.

Given the functional correlation between the loss of CerS2 expression and abnormal polyploidy of liver chromosomes, we performed proteomics analysis on 1‐month‐old WT and CerS2‐depleted liver tissues. Signalling pathway analysis demonstrated that upregulated differential protein expression was primarily distributed in the following signalling pathways: DNA replication, cell cycle and hepatic carcinoma (Figure 3D). Further immunoblotting analyses confirmed that the expressions of these genes, including MCM3/4/5/5 and Mad2 (Figure 3E). In particular, Mad2 is significantly elevated among upregulated cell cycle‐related proteins (Figure 3F). Increased Mad2 expression impairs the function of downstream mitotic kinesin‐like protein 2 (Mklp2) to transfer the chromosome messenger complex (CPC) from the centromere to the central spindle, resulting in incomplete cytokinesis.^10^ We found that Mklp2 protein levels significantly decreased in CerS2‐cKO livers (Figure 3G). Therefore, we hypothesized that CerS2 maintains normal cell division through the MAD2‐MKLP2‐CPC axis.

To further explore the physical function and mechanism of CerS2 in maintaining normal cell division, we successfully constructed CerS2‐knockout cell lines (KO) (Figure S2A–C). Next, we randomly selected certain CerS2‐KO cell lines (KO1 and KO2) to stain with DAPI for phenotype analysis in vitro. The KO1 and KO2 cell lines were consistent with the phenotype of large nuclei in the liver of CerS2‐cKO mice in vivo (Figure 4A,B). Flow‐cytometric analyses were performed to analyse the cell cycle profiles of Ctrl and KO groups (KO1 and KO2) (Figure 4C). Quantitative analysis demonstrated that the proportions of tetraploid (4c) and larger than tetraploid (4c) cells significantly increased in KO groups (Figure 4D). Time‐lapse imaging showed that CerS2 loss resulted in a significant delay during metaphase in mitosis (Figures 4E,F) and a subsequent failure of cell division at different stages, typically accompanied by abnormal membrane blebbing. Altogether, CerS2 deletion in cultured cell lines presented a phenotype consistent with that in vivo.

**FIGURE 4:**
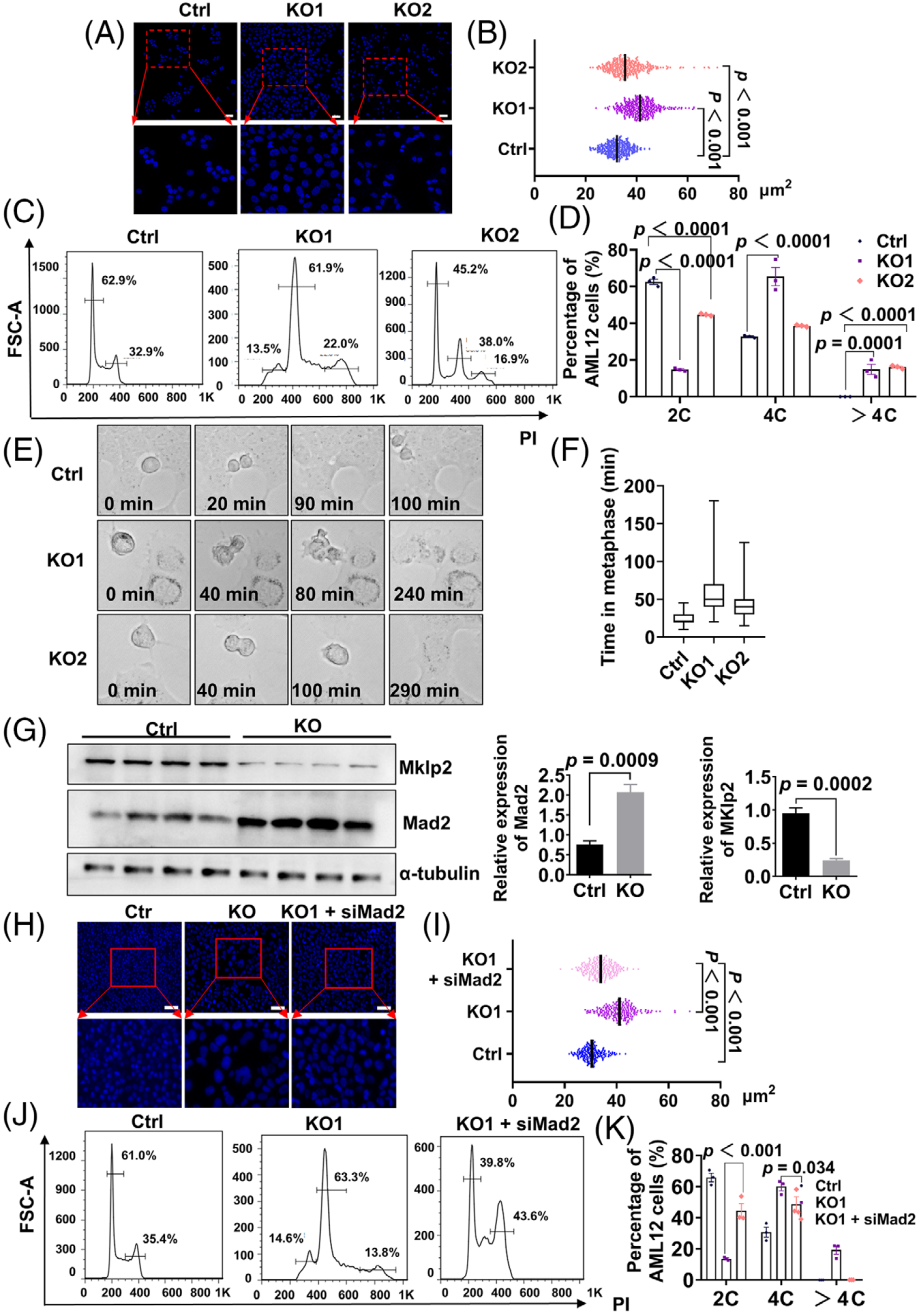
Downregulation of Mad2 alleviates chromosome polyploidization in CerS2‐deficient cell lines. (A) Representative images of nuclei in ctrl, KO1 and KO2 cell lines. The red dotted area on the upper indicates magnification. (B) Statistics of nucleus areas are on the right. Scale bars: 50 μm. (C) The genome ploidies of ctrl, KO1 and KO2 cell lines were characterized by propidium iodide staining, followed by fluorescence‐activated cell sorting. (D) Quantitation of DNA contents from ctrl, KO1and KO2 cell lines. (E) Time‐lapse imaging to analyse the cell cycle of ctrl, KO1 and KO2 cell lines. Results are representative of three independent experiments. KO1 and KO2 represent two CerS2 knockout cell lines. Scale bars: 25 μm. (F) Statistics of metaphase delay time in Ctrl, KO1 and KO2. Results are representative of at least 80 cells from three independent experiments. (G) Western blotting identified Mad2 and Mklp2 expression proteins. (H) Representative images of nuclei in ctrl, KO1 and KO1 + siMad2 cell lines. The red dotted area on the top indicates magnification. (I) Statistics of nucleus areas are on the right. Scale bars: 100 μm. (J) The genome ploidies of ctrl, KO1 and KO1 + siMad2 cell lines were characterized by propidium iodide staining, followed by fluorescence‐activated cell sorting. (K) Quantitation of DNA contents from ctrl, KO1 and KO1 + siMad2 cell lines

To further validate that hypothesis, which is similar to in vivo tests, we verified that the expression of Mad2 and Mklp2 was up‐and downregulated in CerS2‐KO cell lines, respectively (Figure 4G). We next constructed three Mad2 siRNAs (Figure S2D) to explore whether Mad2 knockdown could mitigate chromosome polyploidization induced by CerS2 deletion. DAPI immunostaining demonstrated that the nuclear area of cells in the siRNA transfected group (KO1 + SiMad2) was significantly smaller than that of the KO1 cells (Figure 4H,I). The cell cycle patterns were detected, and there were no octoploid (8c) cells in the KO1 + SiMad2 cells, and that the percentage of tetraploids (4c) significantly decreased (Figure 4J,K). These results indicated that the downregulation of Mad2 can alleviate chromosome polyploidization in CerS2‐deficient cell lines. We next explored whether supplementing sphingolipids with the specific acyl chain length could rescue abnormal chromosome polyploidy induced by CerS2 deletion. Unexpectedly it did not rescue abnormal chromosome polyploidy (Figure S3). This indicates that lipid changes primarily resulted from reflecting abnormal chromosome polyploidy in the CerS2‐knockout liver.

Collectively, these results demonstrate that abnormal chromosome polyploidy caused by CerS2 deletion in vivo and in vitro was primarily due to Mad2 dysregulation during the cell cycle.

## CONFLICT OF INTEREST

Sin Man Lam is an employee of LipidALL Technologies. The other authors declared no conflict of interest.

## Supporting information

Supporting Information

Video S1‐S2
