# Supplementary material for: Hepatic loss of CerS2 induces cell division defects via a mad2‐mediated pathway
Source: Clin Transl Med. 2022 Jan 28;12(1):e712. doi: 10.1002/ctm2.712 (PMC8797468; doi:10.1002/ctm2.712)
Supplement: Supplementary file 1 — Supporting Information [file CTM2-12-e712-s001.docx]

**Supporting Information:**

**Hepatic loss of CerS2 induces cell division defects via a mad2-mediated pathway**

Mingjun Cao^1,2^, Shaohua Zhang^1^, Sin Man Lam^1,3^, Guanghou Shui^1,2*^

^1^State Key Laboratory of Molecular Developmental Biology, Institute of Genetics and Developmental Biology, Chinese Academy of Sciences, Beijing, 100101, China; University of Chinese Academy of Sciences, Beijing, 100101, China.

^2^University of Chinese Academy of Sciences, Beijing, 100101, China. Electronic address: ghshui@genetics.ac.cn.

^3^Lipidall Technologies Company Limited, Changzhou, 213000, China.

*Corresponding author:

Guanghou Shui

Professor, Institute of Genetics and Developmental Biology, Chinese Academy of Sciences

Director, Center for Advanced Technologies

No 1. West Beichen Road, Chaoyang District, Beijing 100101

Tel.: +86 10 6480 7781; Fax: +86 10 6480 6670

Email: ghshui@genetics.ac.cn

1. **Supplementary Materials and methods**

**1.1 Animals**

CerS2 floxed mice (loxp mice) and Alb-Cre tool mice were supplied by Biocytogen and the Model Animal Research Center of Nanjing University, respectively. All mice were maintained under specific pathogen-free (SPF) conditions on a 12 hours light/12 hours dark cycle with continual access to water and a regular rodent chow diet. To generate liver-specific CerS2 knockout mice (cKO mice), loxp mice were crossed with Albumin-Cre transgenic mice to produce offspring with the loxp/+;Alb-cre genotype, after which the loxp/+;Alb-cre mice were mated with loxp/loxp mice. Offspring with the genotype loxp/loxp served as the control group and genotype loxp/loxp;Alb-cre served as the experimental group. All animal experiments were reviewed and approved by the Laboratory Animal Ethics Committee of the Institute of Genetics and Developmental Biology, Chinese Academy of Sciences.

**1.2 Isolation and culture of mouse primary hepatocytes**

Primary hepatocytes were isolated from the livers of loxp and cKO mice via two-step perfusion. The mice were anesthetized via intraperitoneal injection of 4% pentobarbital. After the mice were fully anesthetized, the abdominal cavity was opened to fully expose the inferior vena cava and portal vein, after which the cannula was inserted into the vena cava. The peristaltic pump was turned on, which allowed the perfusion medium I (50 mL, 37˚C, pre-warmed, pH 7.4, no calcium and magnesium HBSS containing 10 mM HEPES and 200 mM EDTA) to perfuse through the liver at 5 mL/min. Once the liver turned pale, the cannulation was successful and we quickly cut the portal vein and perfused it with perfusion medium I for approximately 10 min. We then changed to the fresh perfusion medium Ⅱ (50 mL, pre-warmed, pH 7.4, with calcium and magnesium HBSS containing 0.5 mg/mL collagenase IV and 20 mM HEPES) to perfuse through the liver at 5 mL/min. We then immediately placed the liver into the 10 cm dish containing HBSS with calcium and magnesium on ice and used tweezers to open the membrane of the liver surface and completely release the hepatocytes. The resulting cell suspension was filtered through a 70 mm mesh filter and washed with pre-cooled HBSS three times (centrifuged at 50 g for 3 min at 4℃). The cells were then gently resuspended in 10 mL Dulbecco's Modified Eage Medium: F12 (DMEM/F12, Hyclone) supplemented with 10% fetal bovine serum (FBS, Gibco), 10% human recombinant insulin, human transferrin, and sodium selenite (ITS, Sigma), 20 ug/ml dexamethasone and 1% Penicillin and streptomycin (PS, Gibco), after which they were seeded at a final density of 3-5×10^5^ cells per milliliter. Cells were incubated at 37℃ in 5% CO_2_ for 2 hours, after which the medium was changed for additional culture.

**1.3 Cell line culture and treatment**

AML12 cells were obtained from the Chinese Academy of Sciences (Shanghai, China). AML12 cells were cultured in EMF/F12 supplemented with 10% FBS, 10% ITS, 20 ug/ml dexamethasone, and 1% PS. All cell lines used in the experiments were incubated at 37℃ in 5% CO_2_. For ceramide and sphingomyelin treatment, the procedure was performed as described in previous research^1^. In brief, AML12 cells were seeded into gelatin-coated 6-well plates and cultured in EMF/F12 supplemented with 0.5% FA-free bovine serum albumin, 10% ITS, 20 ug/ml dexamethasone. The cells were then permeabilized with 20 μg/mL digitonin for 5 min and treated for 48h with 10 µM ceramide (d18:1/16:0), C24-ceramide (d18:1/24:1), 3µM spingomyelin (d18:1/17:0), and shingomyelin (d18:1/24:0), respectively. The ceramide and sphingomyelin were solubilized in 2% dodecane/98% ethanol solution.

**1.4 Construction of CerS2 knockout cell lines**

The CerS2 knockout cell lines were constructed with the CRISPR-Cas9 system, and the timeline and overview of experiments proceeded as follows. CerS2-sgRNAs were performed using the CRISPR Design Tool (<http://tools.genomeengineering.org>), while CerS2-sgRNA guide sequences were cloned into an expression plasmid bearing both sgRNA scaffold backbone (BB) and Cas9, pSpCas9 (BB). The resulting plasmid was annotated as pSpCas9 (CerS2-sgRNA), which was completed and sequence-verified. Finally, AML12 cells transfected with the plasmids were clonally expanded to obtain CerS2 knockout cell lines.

**1.5 Isolation of mouse hepatocytes nuclei**

The excised liver was cleaned of extraneous tissues and weighed. The entire liver was kept on ice with 4 mL of ice-cold buffer A (250 mM sucrose, 5 mM MgCl_2_, and 10 mM Tris–HCl [pH 7.4]) for each 0.5g of the liver. The mass of disrupted cells was centrifuged at 600g for 10 min at 4℃. The pellet was then gently resuspended in 14 mL of ice-cold buffer A and centrifuged at 600g for 10 min at 4℃, after which the process was repeated. This crude nuclei pellet was resuspended in nine volumes of ice-cold buffer B (2.0 M sucrose, 1 mM MgCl_2_, and 10 mM Tris–HCl [pH 7.4]), well mixed, distributed into microtubes of 1.5 mL, and centrifuged at 16,000g at 4℃ for 30 min. The crude nuclei were separated into two layers, and the white pellet of isolated nuclei was on the bottom of the tube. The pellet of isolated nuclei was maintained at -80℃ for subsequent analysis.

**1.6 Western blotting**

AML12 cells, mice liver tissues, or hepatocyte nuclei were harvested and lysed with the lysis buffer (Beyotime). Protein concentrations were measured using the Pierce™ BCA Protein Assay Kit (Thermo Fisher). The protein samples were then denatured at 98°C for 8-12 minutes and resolved using sodium dodecyl sulfate-polyacrylamide gel electrophoresis (10% or 12% SDS-PAGE gel). After transferring the proteins onto polyvinylidene fluoride membranes (0.45 µm pore size, Merk Millipore), they were incubated with a blocking buffer (5% nonfat milk diluted in Tris-buffered saline containing 0.1% Tween-20) for 1 hour at room temperature. The membranes were then incubated with the primary antibodies in blocking solutions at 4˚C overnight before detection with HRP-conjugated secondary antibodies (ZSGB-BIO). Blot signals were visualized using “SuperSingnal™ West Dura Extended Duration Substrate,” which was purchased from Thermo Fisher. Finally, we performed grayscale analysis with ImageJ (National Institutes of Health, Java image processing software). The following primary antibodies were used: anti-CerS2 (1;200, Rabbit, Sigma), anti-α-tubulin (1:5000, Rabbit, abcam), anti-MCM3 (1:1000, mouse, Santa Cruz), anti-MCM4 (1:1000, mouse, Santa Cruz), anti-MCM5 (1:1000, mouse, Santa Cruz), anti-MCM7 (1:1000, mouse, Santa Cruz), anti-Mad2 (1:300, mouse, Santa Cruz), anti-Mklp2 (1:300, mouse, Santa Cruz), and anti-β-actin (1:5000, Rabbit, abcam).

**1.7 Histological analysis**

Livers were collected and fixed in 4% paraformaldehyde (PFA) for at least 12 hours. After paraffin-embedding and a serial section for 5 μm thickness, H&E staining was performed.

**1.8 Immunofluorescence staining**

The isolated liver was fixed with 4% PFA for at least 12 hours and then prepared for paraffin-embedding and sectioning. After dewaxing and rehydration, the tissue sections were washed and the membranes were cut. Sections were then blocked with 5% goat serum in Tris-buffered saline containing 0.1% Tween-20 (TBST) for 1 hour at room temperature, after which the sections were incubated with primary antibodies overnight at 4°C. After washing, the samples were incubated with secondary antibodies at 37°C for 1 hour. After washing them again, 4′,6-diamidino-2-phenylindole dihydrochloride (DAPI) was counterstained to probe the DNA for 15 min. Finally, the sections were mounted with mounting medium and imaged with a confocal laser scanning microscope (Zeiss LSM780). The primary antibodies used for immunostaining included anti-β-catenin (1:200, #8456, CST), anti-PCNA (1:200, sc-56, Santa Cruz), anti-Ki-67 (1:200, ab15580, abcam), and anti-phospho-H3S10 (1:200, 9701S, CST).

**1.9 Oil Red O staining of mouse liver sections**

Freshly collected liver tissue of control (loxp) and CerS2-cKO mice fed in chow diets were embedded in OCT (Tissue-Tek) and immediately froze with liquid nitrogen. After the consecutive 10-µm-thick sectioning, the liver samples were fixed immediately with ice-cold 4% (w/v) paraformaldehyde (PFA) dissolved in PBS (pH 7.4) for 10 mins. After rinsing three time, slides were dehydrated with 60% isopropyl alcohol and then immersed by Oil Red O staining solution at room temperature for 15 mins. Next step is nucleus counterstaining with Mayer′s hematoxylin for 10 second and thereafter bluing the stain in PBS (pH 7.2) for 5 min.

**1.10 Flow cytometry analysis**

Freshly isolated hepatocytes were fixed in ice-cold 70% ethanol overnight at -20˚C and then stained with 5 mg/ml propidium iodide supplemented with 250 mg/ml RNase A in PBS. The DNA contents of 30,000 cells per sample were analyzed with a BD FACS Calibu flow cytometer (BD Biosciences, San Jose CA, USA) and Flowjo analysis software 7.0 (TreeStar, Ashland OR, USA).

**1.11 Time-lapse imaging**

Primary hepatocytes were isolated and cultured in costar 6-well tissue culture plates (#3516, Corning, Oneonta NY, USA). Two hours later, once the hepatocytes were attached to the plate, the medium was replaced by EMF/F12 supplemented with 10% FBS, 10% ITS, 20ug/ml dexamethasone, and 1% PS: iMEF conditional medium (1:1) and incubated at 37℃ in 5% CO_2_ to plate for 24 hours. Hoechst 33342 (Invitrogen) was added to the medium with a final concentration of 50 μg/mL to label the hepatocytes nuclei before time-lapse imaging. Fresh mediums were replaced 1 hour later. Primary hepatocytes were imaged at 10 min intervals for 30 hours with Zeiss LSM780 confocal microscope at 37℃ in a 5% CO_2_ atmosphere.

**1.12 LC-MS/MS analysis**

Lipidomics analysis was performed on the Exion UPLC system combined with the QTRAP 6500 Plus system (SCIEX)^2^. he positive ion mode was used to and quantify levels of ceramide, sphingomyelin, glucoseceramide (GLuCer), lactoseramide (LacCer), sphingosine 1-phosphate (S1P), and sphingosine (Sph). Individual lipids were quantitated relative to their respective internal standards.

**1.13 Proteomic analysis**

Proteomic analysis was performed as described in previous research^3^. In brief, the mouse liver tissue proteins were extracted with 8M urea (containing a 1% protease inhibitor). Then, 200 μg protein was obtained for enzymatic hydrolysis in the solution. We used Sep-Pak Pillars (Waters, 186004619) for desalination; after the desalted sample was vacuum-dried and labeled with Tandem Mass Tag (TMT, Thermo, Waltham, MA). We then used high-pH reversed-phase chromatography followed by nano LC-MS/MS (Thermo, Q-Exactive, 14017289) analysis. The conditions were as follows: XBridgeTM BEH300 C18 column (Waters, MA), Phase A mobile phase: 100% H2O (PH = 10), Phase B mobile phase: 98% acetonitrile, 2% H2O (PH = 10).

**1.14 Statistical analysis**

All experiments were repeated at least three times; data are presented as mean ± SEM.

A Student’s t-test was used to compared two groups of data, while more than two groups were compared using one-way ANOVA. Immunoblot and immunofluorescence results used Image J software for grey value and area statistics, respectively. Significance was defined as * *P* < 0.05, ** *P* < 0.01, *** *P* < 0.001, and **** *P* < 0.0001.

**Supplementary References**

1. Matsuzaka T, Kuba M, Koyasu S, et al. Hepatocyte ELOVL Fatty Acid Elongase 6 Determines Ceramide Acyl-Chain Length and Hepatic Insulin Sensitivity in Mice. *Hepatology.* 2020;71(5):1609-1625.

2. Wang X, Lam SM, Cao M, et al. Localized increases in CEPT1 and ATGL elevate plasmalogen phosphatidylcholines in HDLs contributing to atheroprotective lipid profiles in hyperglycemic GCK-MODY. *Redox Biol.* 2021;40:101855.

3. Yi M, Ma Y, Zhu S, et al. Comparative proteomic analysis identifies biomarkers for renal aging. *Aging (Albany NY).* 2020;12(21):21890-21903.

**Supplementary Figures**

**
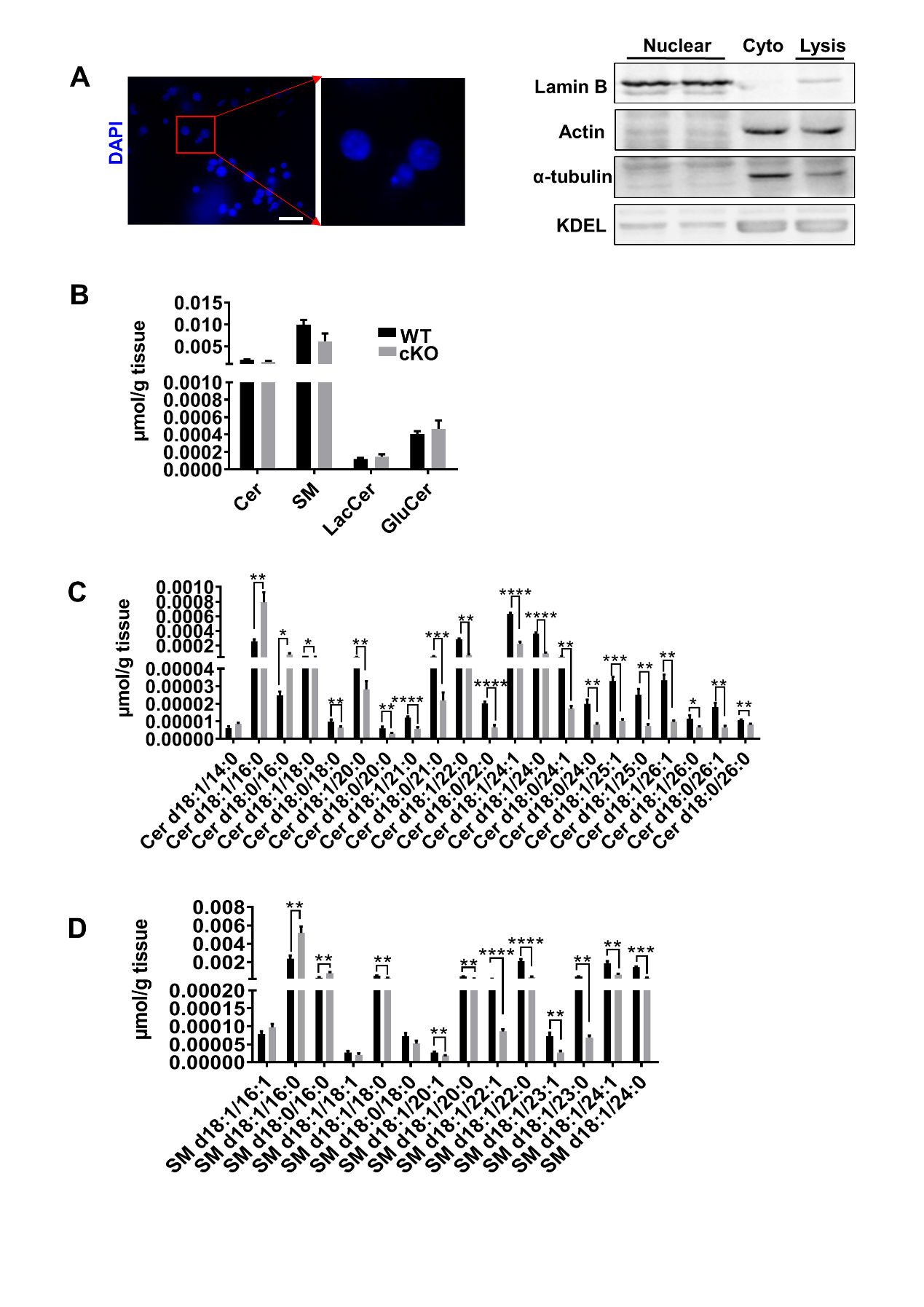
**

Figure S1 Effect of CerS2 on sphingolipid metabolic pathways in hepatocyte nuclei. (A) Representative images of nuclei in the hepatocytes of WT mice. The red dotted area on the left indicates magnification. Scale bars: 50 µm. (B) Western blot analyses of Lamin B, a marker of the nuclear; actin and α-tubulin, a marker of the cytoplasm; KDEL, a marker of the endoplasmic reticulum. (C) The total levels of Cer, SM, LacCer, and GluCer were analyzed by LC-MS/MS in WT and cKO mice livers at 1 month. (D-F) Cer, and SM with specific chain length levels were analyzed by LC-MS/MS. n = 4 mice per group. Abbreviations: Ceramide, Cer; sphingomyelin, SM; Glucoseceramide, GLuCer; Lactoseramide, LacCer. *, *p* < 0.05; **, *p* < 0.01; ***, *p* < 0.001 and ****, *p*< 0.0001.


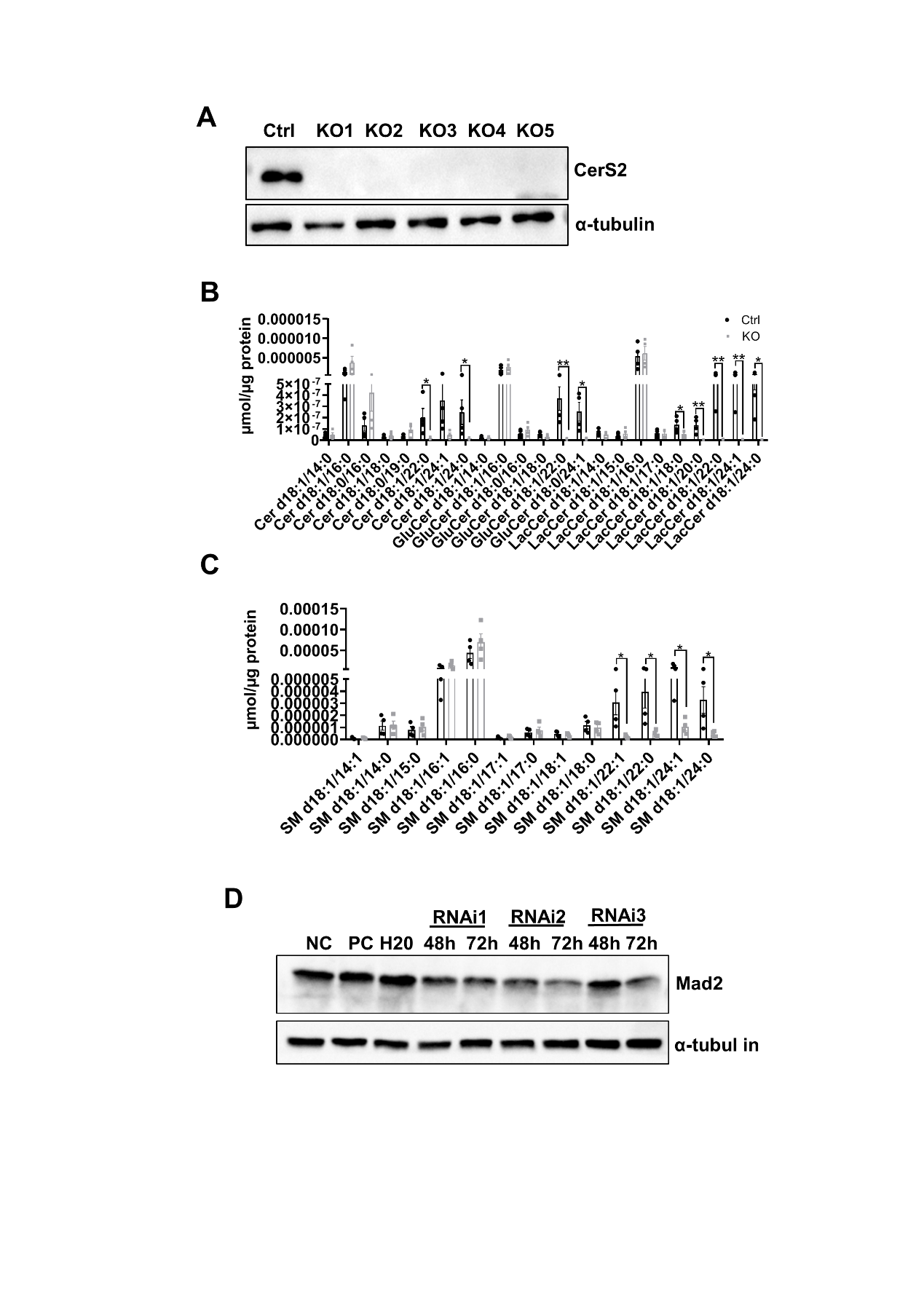


Figure S2 Generation of CerS2 knockout cell line. (A) Western blotting analyses of CerS2 expression in CerS2 knockout AML12 cell line normalized to α-tubulin, and only five monoclones were shown. (B-C) Cer, GluCer, LacCer, and SM with specific chain length levels were analyzed by LC-MS/MS in Ctrl and KO cell lines. (D) Western blot analyses of Mad2 expression in AML12 cell line after 48 and 72 hours of siMad2 treatment normalized to α-tubulin.

**
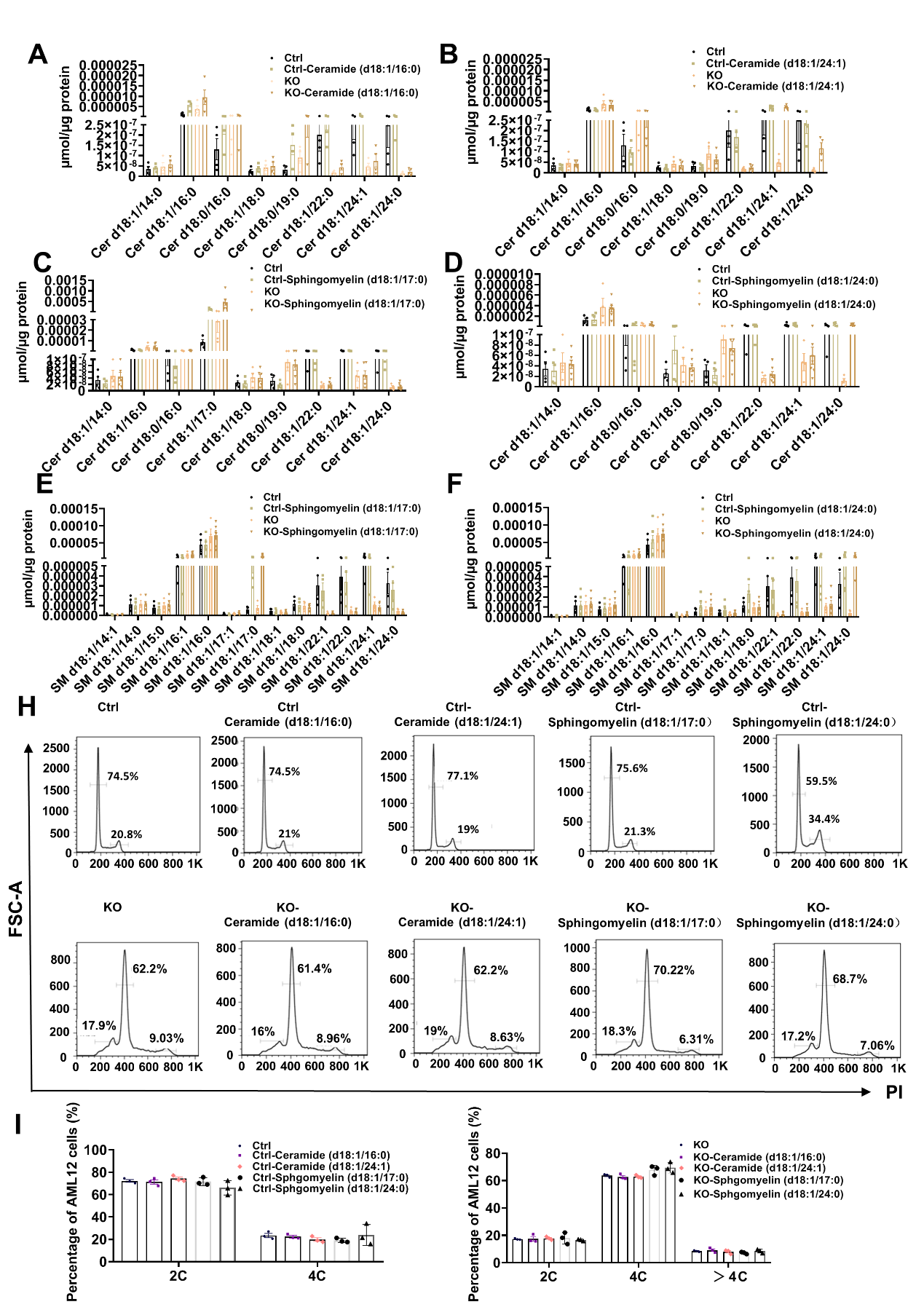
**

Figure S3. Sphingolipids-based levels and polyploids in CerS2 knockout cell lines after treatment with ceramide and sphingomyelin with specific acyl chain length. (A-F) Levels of Cer with specific chain length. LC-MS/MS analyses were performed in Ctrl and KO cell lines after treatment with ceramide- and sphingomyelin-specific acyl chain length. (H) The genome ploidies in both Ctrl and KO cell lines after treatment with ceramide- and sphingomyelin- specific acyl chain length. (I) Quantification of DNA contents from Ctrl and KO cell lines after incubation treatment.
