# Supplementary figures and images for: Hepatic loss of CerS2 induces cell division defects via a mad2‐mediated pathway
Source: Clin Transl Med. 2022 Jan 28;12(1):e712. doi: 10.1002/ctm2.712 (PMC8797468; doi:10.1002/ctm2.712)

## Slide 1
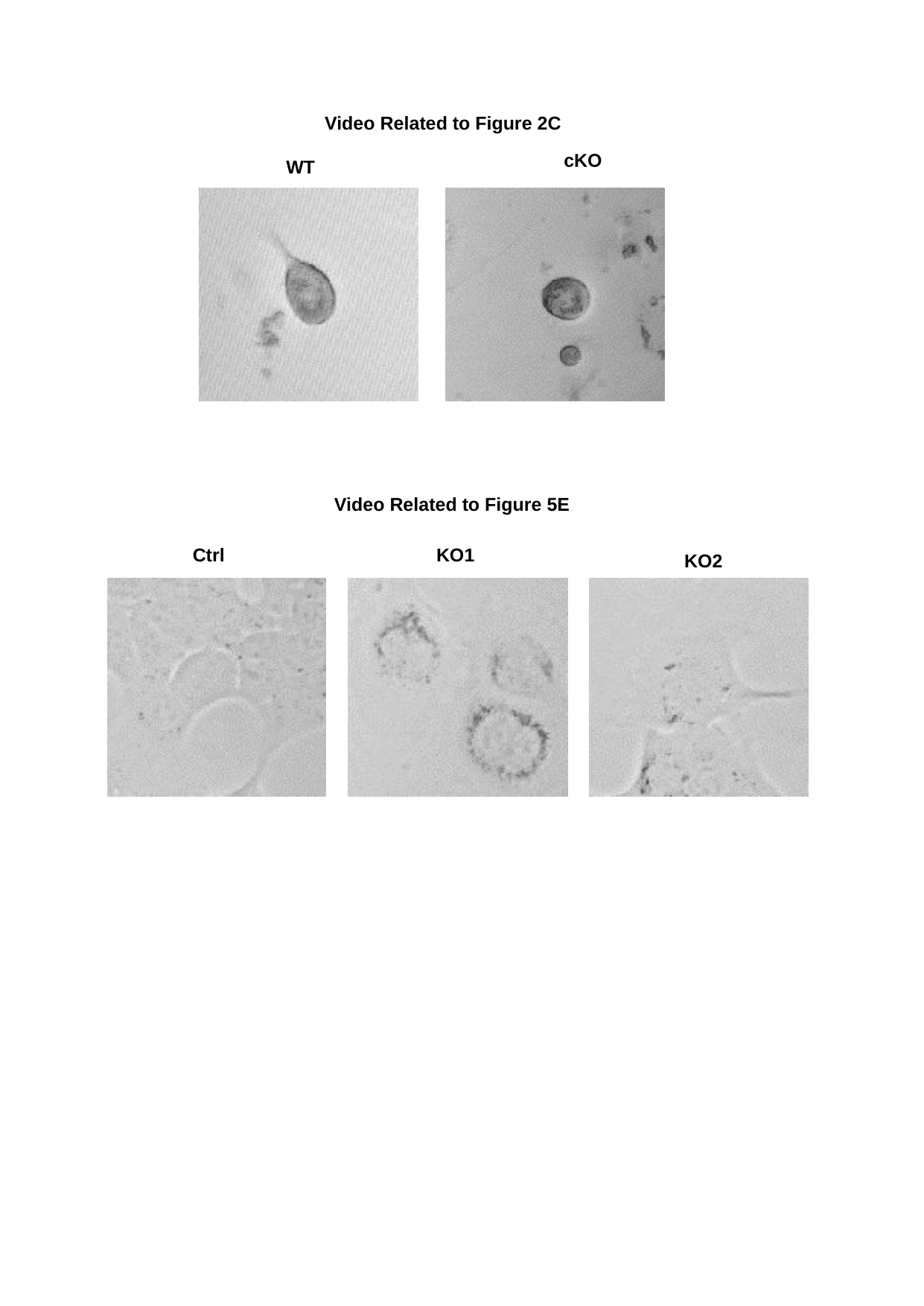

Video Related to Figure 2C
cKO
WT
Video Related to Figure 5E
KO1
Ctrl
KO2

Supplement: Supplementary file 2 — Video S1‐S2 [file CTM2-12-e712-s002.pptx]
